# Editorial for the special section: Obesity in Asia

**DOI:** 10.1002/osp4.662

**Published:** 2023-02-10

**Authors:** David B. Sarwer

**Affiliations:** ^1^ Center for Obesity Research and Education College of Public Health Temple University Philadelphia Pennsylvania USA

As readers of the journal know all‐too‐well, the worldwide obesity problem continues to grow largely unchecked. Prevalence rate projections from decades ago, which many believed forecasted a worst case scenario, have been quite accurate. Unsurprisingly, rates of several obesity‐related co‐morbidities have followed the trajectory. Projections for the future are sobering. By 2030, more than one billion persons around the world may be living with obesity.

Rates of obesity in low and middle‐income countries continue to rise rapidly; in some areas of the world the rates are now comparable to what was previously reserved for high income countries. The financial costs associated with the disease pose a legitimate threat to economic growth if not stability. The costs at the individual level are deeply troubling with persons with obesity losing 4–8 years of disease free years of their lives, depending on their body mass index. We clearly have much to do, and on multiple levels, if we expect to change this trajectory.

*Obesity Science and Practice* was first published in 2015. One of the main goals in creating the journal was to provide a home for the research and scholarship from the increasing number of multidisciplinary professionals from around the globe. As the journal has grown and matured, the international focus of the journal has increased. The number of articles from outside of North America increases with each year. When new members of the Editorial Board are named, as will happen in 2023, the addition of accomplished professionals from around the world is prioritized.

To further our international reach, this issue of the journal includes a special section of articles from research teams in Asia. The section includes four original articles and one review paper. The articles report on the relationship of obesity to comorbidities such as cardiovascular disease and gallbladder disease, dietary contributions to obesity in adolescents and adults, and the relationship of obesity to the effectiveness of the SARS‐CoV‐2 vaccine. I hope you enjoy these articles and find them useful to your own work.

Rates of obesity in low and middle‐income countries continue to rise rapidly; in some areas of the world the rates are now comparable to what was previously reserved for high income countries. The financial costs associated with the disease pose a legitimate threat to economic growth if not stability. The costs at the individual level are deeply troubling with persons with obesity losing 4–8 years of disease free years of their lives, depending on their body mass index. We clearly have much to do, and on multiple levels, if we expect to change this trajectory.

With this in mind, our next special issue will likely focus on emerging treatments. Look out for the call for submissions in the near future. Even in advance of that call, keep the journal in mind as a home for your scholarship and to inform and educate all of us on the most recent developments to understand and address the global obesity problem.

## CONFLICT OF INTEREST STATEMENT

Dr. Sarwer currently has grant funding from the National Institute of Diabetes, Digestive, and Kidney Disease, National Institute of Dental and Craniofacial Research, and the Department of Defense. He has consulting relationships with Ethicon, NovoNordisk, and Twenty30 Health.

